# Disease modifying treatment trials in Parkinson’s disease: how to balance expectations and interests of patients, physicians and industry partners?

**DOI:** 10.1186/s42466-020-00076-y

**Published:** 2020-11-02

**Authors:** D. Berg, K. Eggert, B. Haslinger, J. Kassubek, B. Mollenhauer, K. Reetz, A. Rogge, E. Schaeffer, L. Tönges, K. E. Zeuner

**Affiliations:** 1 Department of Neurology, Christian-Albrecht University of Kiel, Arnold-Heller-Straße 3, 24105 Kiel, Germany; 2 Department of Neurology, Philipps-University of Marburg, Marburg, Germany; 3 Department of Neurology, TU-Muenchen, Munich, Germany; 4 Department of Neurology, University of Ulm, RKU, Ulm, Germany; 5 Paracelsus Klinik, Kassel, Germany; 6 Department of Neurology, RWTH Aachen University, Aachen, Germany; 7 Institute for Experimental Medicine, Medical Ethics, Christian-Albrecht University of Kiel, Kiel, Germany; 8 Department of Neurology, St. Josef-Hospital, Ruhr University, Bochum, Germany

**Keywords:** Parkinson’s Disease, Disease modifying treatments, Clinical trials, Ethical challenges

## Abstract

**Background:**

The advent of therapeutic strategies designed to modify the disease course in Parkinson’s disease has raised great expectations in the currently conducted clinical trials. However, we see ethical challenges in the cooperation of industry and clinical partners, specifically evident in the way recruitment is performed.

We here discuss the different positions and challenges of all involved to set the stage for a study and recruitment culture taking into account the expectations of all: (i) patients and their caregivers, ready to take the considerable burden of clinical trials in hope for the development of disease-modifying treatments; (ii) physicians and study nurses, obligated to the patients’ well-being and benefit who accompany and supervise patients closely as basis for the performance of elaborate clinical trials (iii) industrial partners, investing years of efforts and finances to develop new treatments.

**Conclusions:**

We conclude that the current competitive race for enrollment in clinical studies in PD is challenging the primary goal to ensure patients’ benefit and formulate requests to the industrial partners to encounter these concerns.

## Background

Common neurodegenerative diseases like Alzheimer’s (AD) and Parkinson’s disease (PD) are characterized by their progressive nature and so far, there is no cure and has been only little hope for disease course modulating therapies. Advances in the understanding of the underlying pathologies and disease progression have led to enormous research efforts to target factors associated with pathophysiology and progression. As a result, completely new approaches have been developed ranging from therapies interacting with metabolic (e.g. enzymatic) pathways to immune and stem cell therapies. Long prodromal periods and the slowly progressive nature of the common neurodegenerative diseases require that disease course modulating therapies are optimally initiated as early as possible, despite the diagnostic difficulties at that stage of the disease – and it is hoped that progression of the neurodegenerative process may be slowed down or even stopped before severe symptoms occur.

Whereas in AD disease course addressing therapeutic strategies have been tested for many years already, disease modulating clinical trials for PD have failed in the past and newer strategies only recently emerged. Naturally, we face challenges with these current and upcoming clinical trials in PD, which are matter of ongoing discussions [1]. However, besides scientific, organizational and economic aspects, important ethical concerns should be considered.

We here want to discuss ethical challenges of competitive recruitment in clinical trials, considering positions of patients, caregivers, physicians and study nurses, as well as industrial partners. We derive requests to our industrial partners to meet the overall aim of patient well-being in clinical trials.

## Main text

To improve the situation for the parties involved we here would like to point out one ethical aspect that has already been described almost 15 years ago [2], but has not changed since and seems still of great relevance: Large numbers of (often world-wide) centers are initiated with a competitive recruitment or even parallel recruitment to multiple competing studies with immanent concerns of patient safety. But what happens to those centers who completed all the labor-intensive preparations (which usually includes many different departments such as neurology, ethics, legal office, administration, pharmacy, neuroradiology, etc.), whose patients kept waiting patiently and full of hope but then could not be recruited into the clinical trial because others were just a little bit faster? In our experience it has become a common habit to predate recruitment stops several times, leaving some centers with the recruitment of even none or very few patients.

To understand this challenge the situation of each group involved needs to be appreciated.

### Patients and their relatives/caregivers

Patients and their relatives and especially possible study participants are well educated nowadays and have access to several media. Hence, they are well informed about their disease and the efficacy and disadvantages of the currently available medication. At the same time, they realize that there is no cure for the progressing, life changing disease including motor disability and non-motor symptoms. With access to campaigns like the Fox Trial Finder [3] and others, patients realize opportunities to be part of disease modifying clinical trials. Even if the chance to escape the predestined course is small, many of the affected subjects are willing to undergo severe strains including long distances of travelling and invasive assessments. Moreover, many of the recent studies claiming to modify disease progression demand an inclusion of de novo PD patients, meaning that patients have to wait to be enrolled in the study without taking symptomatic medication for months, followed by an abstinence of symptomatic therapy for the course of the trial, knowing that they may only receive placebo. This huge burden for patients, the scientific justification for this study design and its ethical concerns are not sufficiently scrutinized.

### Physicians and study nurses

Many centers who are involved in the treatment of neurodegenerative diseases have followed their patients for many years and have built up a close, professional relationship with them and their relatives. This relationship is the basis that new treatment options can be tested and implemented in the future.

### Industrial partners

Years of effort - including huge financial investment - have been put into the development of the new treatment, and setbacks, new approaches, strategic planning and careful design of the extensive clinical study had to be coped with. Now, as the trial is approved by the respective authorities the race starts – with a different pace. The major aim now seems to be to recruit fast, an aim that seems only to be accomplishable by competitive recruitment.

### Consequences

Besides the fact that conduction of extensive, complex studies gets better the more individuals are enrolled per site questioning the enrollment of single or very few patients per center, the ethical implications associated with the stirring up of hope should always be kept in mind. The fact that many patients are kept without medication as they themselves want to wait to be enrolled aggrandizes the ethical problem. Moreover, the fact that only the fastest centers get the opportunity to enroll patients entails the danger of the bias to have the majority of patients recruited in countries with less strict public, administrative, and ethical review boards.

As physicians we are first of all obligated to our patients’ benefit. We are dedicated to alleviating their physical and psychological burden, to seek realistic ways of encouragement and to develop perspectives to live a meaningful life with the disease. Hence, we are also deeply interested in new and better treatment strategies and we are willing to contribute to the best of our capabilities to the establishment of promising therapies [4].

The current desperately competitive race for enrollment in clinical studies, however, is not reconcilable with our primary obligation to serve our patients and has to be labelled as a serious confounding factor for the patient-physician relationship and for the motivation to participate in future studies.

Knowing that enrollment is always limited and that only an approved number of individuals can participate in clinical trials we sincerely request our industrial partners to

1. Seek communication with medical professional to realistically plan recruitment strategies
2. Set up realistic and transparent recruitment plans
3. Re-evaluate study designs with questionable scientific rationales which impose a huge burden on patients (particularly de novo study designs)
4. Stick to these plans giving all initiated centers the chance to enroll those who had been waiting and are willing to devote themselves in predefined but binding timeframes
5. Provide transparent screening criteria and optimize communication with CROs as well as official reference labs and imaging centers.
6. Create a neutral ombudsman for the conduct of clinical pharmacological studies for patients, caregivers, medical staff and industry partners

## Conclusions

The currently performed strategies for competitive recruitment in clinical trials in PD result in considerable ethical concerns, including a great burden for PD patients and a potential impairment of patient-physician relationship. If the formulated requests were fulfilled, the realization and performance of disease modifying therapeutic studies would benefit substantially and thereby accelerate the speed to find a cure for PD.

## Data Availability

Not applicable.

## Abbreviations

AD: Alzheimer’s Disease
PD: Parkinson’s Disease

## References

[1] Sherer TB, Chowdhury S, Peabody K, Brooks DW (2012). Overcoming obstacles in Parkinson’s disease. Movement Disorders.

[2] Caulfield T (2005). Legal and ethical issues associated with patient recruitment in clinical trials: The case of competitive enrolment. Health Law Rev..

[3] Chowdhury S, Meunier CC, Cappelletti L, Sherer TB (2014). Improving patient participation in Parkinson‘s clinical trials: The experience of the Michael J fox Foundation. Clinical Investigation..

[4] Miller FG, Rosenstein DL, DeRenzo EG (1998). Professional integrity in clinical research. JAMA.

